# Glutathione prevents chronic oscillating glucose intake-induced β-cell dedifferentiation and failure

**DOI:** 10.1038/s41419-019-1552-y

**Published:** 2019-04-11

**Authors:** Jitai Zhang, Hui An, Kaidi Ni, Bin Chen, Hui Li, Yanqin Li, Guilian Sheng, Chuanzan Zhou, Mengzhen Xie, Saijing Chen, Tong Zhou, Gaoxiong Yang, Xiufang Chen, Gaojun Wu, Shengwei Jin, Ming Li

**Affiliations:** 1 Cardiac Regeneration Research Institute, Wenzhou Medical University, Wenzhou, China; 2 Department of Medical Ultrasound, The First Affiliated Hospital of Wenzhou Medical University, Wenzhou, China; 3 Department of Gynecology and Obstetrics, The Second Affiliated Hospital of Wenzhou Medical University, Wenzhou, China; 4 Department of Biochemistry, School of Basic Medical Science, Whenzhou Medical University, Wenzhou, China; 5 The First Affiliated Hospital of Wenzhou Medical University, Wenzhou, China; 6 Department of Anesthesia and Critical Care, The Second Affiliated Hospital of Wenzhou Medical University, Wenzhou, China

## Abstract

Modern lifestyles have altered diet and metabolic homeostasis, with increased sugar intake, glycemic index, and prediabetes. A strong positive correlation between sugar consumption and diabetic incidence is revealed, but the underlying mechanisms remain obscure. Here we show that oral intake of long-term oscillating glucose (LOsG) (4 times/day) for 38 days, which produces physiological glycemic variability in rats, can lead to β-cells gaining metabolic memory in reactive oxygen species (ROS) stress. This stress leads to suppression of forkhead box O1 (FoxO1) signaling and subsequent upregulation of thioredoxin interacting protein, inhibition of insulin and SOD-2 expression, re-expression of Neurog3, and β-cell dedifferentiation and functional failure. LOsG-treated animals develop prediabetes exhibiting hypoinsulinemia and glucose intolerance. Dynamic and timely administration of antioxidant glutathione prevents LOsG/ROS-induced β-cell failure and prediabetes. We propose that ROS stress is the initial step in LOsG-inducing prediabetes. Manipulating glutathione-related pathways may offer novel options for preventing the occurrence and development of diabetes.

## Introduction

Pancreatic β-cell is the primary regulatory center that controls the primary-fuel glucose homeostasis. Excessive nutrient intake relative to energy expenditure has fueled a dramatic increase in the incidence of diabetes^1^, which is mainly due to a relentless decline in β-cell function. It was estimated that the population with diabeties worldwide would increase from 451 million people in 2017 to 693 million by 2045^2^. Meta-analyses have indicated a strong relationship between sugar consumption and obesity, diabetes, and the metabolic syndrome^3^. However, no definitive studies show an obvious relationship between the intake of total carbohydrates and glycemic control that can lead to type 2 diabetes (T2DM)^4,5^. Many people today usually not only have more sugars but also eat more frequently. This results in oscillating glycemia, which challenges the energy metabolic homeostasis. Oscillating glucose (OsG), leading to glycemia fluctuation every 6 h for 24 h, is more deleterious to endothelial function and oxidative stress than stable glucose in normal and T2DM patients^6^. β-Cell is vulnerable to reactive oxygen species (ROS)^7,8^. T2DM is associated with fluctuating hyperglycemia despite optimal medical treatment^8,9^. Interestingly, diabetes attenuates the protective ability of females, who are more in favor of sweet foods, against the development of cardiac diseases and nephropathy^10^. In light of these findings, we postulated that long-term fluctuating glycemia, especially after extra carbohydrate intake, continuously and waveringly generates extra ROS, which in turn damages the pancreatic β-cells. Dynamic and timely administration of antioxidant glutathione (GSH) can block the glucose/ROS-induced β-cell damages.

## Methods

### Animals and ethics statement

Previous studies have shown that diabetes attenuates the protective ability of females, who are more in favor of sweet foods, against the development of cardiac diseases and nephropathy^10^. We therefore used female animals in our study. Sprague–Dawley female rats with a body weight of 200 ± 10 g (mean ± SD) were purchased from the Chinese Academy of Medical Sciences (Shanghai, China). Rats were acclimatized to a controlled environment of 22 ± 1 °C temperature and a 12-h light/dark cycle, with free access to food and water, for 2 weeks prior to the experiments. Rats received a regular diet with 49.39% of energy derived from carbohydrates, 31.67% from protein, and 18.94% from fat. The diet composition is listed in Table 1. The research protocol was approved by the Institutional Animal Care and Use Committee of Wenzhou Medical University, China. All experiments were conducted according to the guidelines of the committee.

**Table 1:** Composition of rat regular diet

Ingredient	Percentage (%)
Indian corn	40
Wheat bran	33
Soybean	10
Fish flour	12.5
Yolk flour	0.5
Milk powder	1.5
Shells flour	1
Calcium phosphate	1
Mulvital	0.035
Minor element	0.15
Sodium chloride	0.35
Total	100

### Metabolic studies

Animals were given either 6 g/kg glucose or distilled water (2 mL/100 g body weight, *n* = 10 for all groups) by gavage every 6 h (at 06:20, 12:20, 18:20, and 24:20 h) for 38 days, defined as the long-term oscillating glucose (LOsG) or sham group. To test the hypothesis that a disorder of ROS homeostasis is induced by OsG-produced oxidative stress, an antioxidant, GSH (50 mg/kg/ 6 h), was subcutaneously injected into animals with LOsG challenge as described above, defined as the LOsG.TdGSH group (*n* = 10). We measured body weight every day for 38 days. Overnight (22:00–10:00 h) fasting blood glucose (FBG) was measured with an Accu-Chek^®^ reflectance meter with Accu-Chek active test strips (Roche Diabetes Care, Mannheim, Germany) on experimental days P7, P14, P21, P28, and P38. On experimental day P38, the oral glucose tolerance test (OGTT) was performed on overnight-fasted rats. A glucose load (2 g/kg body weight) was given to each rat orally with a feeding syringe. Blood samples were collected from the tail vein at 0, 30, 60, 90, and 120 min of glucose administration, and the blood glucose level was determined using a glucometer (Accu-Chek Active; Roche, Mannheim, Germany).

On experimental day P38, we measured plasma insulin using the Rat INS ELISA Kit (Mlbio, Shanghai, China) according to the instructions. Briefly, 100 μl of enzyme conjugate was added to the wells containing 50 μl of standard or plasma, covered with an adhesive strip and incubated for 60 min at 37 °C. The microtiter plate was washed four times with a wash buffer (1×). Then substrate A (50 μl) and substrate B (50 μl) were added to each well, gently mixed, and incubated for 15 min at 37 °C in dark. Fifty microliters of stop solution was added to each well and the optical density at 450 nm determined using a microtiter plate reader (Liuyi, Beijing, China) within 15 min.

For plasma biochemical profiles analysis, overnight fasting blood samples were obtained and centrifuged at 3000 r.p.m. for 10 min. Samples were frozen at −80 °C until use. Total cholesterol, triglyceride, high-density lipoprotein, low-density lipoprotein, and lipase were analyzed using the ADVIA 2400 Clinical Chemistry System from Diasys Diagnostics Systems GmbH (Frankfurt, Germany).

### Fresh isolation of white blood cells and ROS detection

Blood samples were collected from rats before and 1 h after glucose challenge (gavage of 2 g/kg body weight). All steps of white blood cell (WBC) preparation and ROS detection were performed as described previously^11–13^ with modifications. In brief, peripheral blood samples (300 μl) for WBC isolation were collected in tubes with heparin. The WBC population was separated by hypotonic lysis of erythrocytes with red blood cell lysate (Solarbio, Beijing, China). Isolated WBCs were diluted in PBS (1×) at a final concentration of 2 × 10^6^ /ml. The viability of WBCs was checked by flow cytometry and it was consistently >95%.

WBC ROS was measured using 2,7-dichlorodihydrofluorescein diacetate (DCFH-DA) (2,7-dichlorodi-hydrofluorescein diacetate; Phygene, Fuzhou, China), which can readily enter cells and be cleaved by esterase to yield DCFH, a polar, nonfluorescent product. Cell ROS can promote the oxidation of DCFH to produce the fluorescent product, dichlorofluorescein. Cells were collected and then incubated with serum-free DMEM containing 10 µM DCFH-DA for 30 min at 37 ℃. Blank controls were set, in which DCFH-DA incubation was omitted. After incubation, cells were washed with PBS twice, re-suspended, and immediately submitted to flow cytometric analysis using a BD Accuri C6 Plus flow cytometer (Becton Dickinson, Mountain View, CA, USA). As we were interested in studying the changes in ROS production in peripheral blood cells, WBC (5 × 10^4^ cells/sample) ROS as a whole was analyzed with the BD Accuri C6 Plus software.

### Pancreatic ROS detection

On experimental day P38, pancreas was collected. Tissue ROS detection was performed as described^14^. The ROS indicator oxidative fluorescent dye dihydroethidium (DHE; Beyotime Institute of Biotechnology, Shanghai, China) staining was used to detect the ROS generation in situ. Harvested pancreas tissue sections (5 μm) were stained with 5 μM DHE at room temperature for 60 min in the dark according to the instructions provided by the manufacturer. The ROS generation was detected with a Leica fluorescence microscope (Leica DM6000B) using Leica LAS X software, and quantitative analysis was performed with ImageJ software (version 1.60; National Institutes of Health, Bethesda, MD, USA).

### Quantitative RT-PCR

Pancreatic tissues were collected on experimental day P38. RNA was extracted from snap frozen tissue and real-time qRT-PCR performed as described previously^15^. Total RNA was extracted from with Trizol reagent (Beyotime, Shanghai, China) according to the manufacturer’s instructions. One μg of RNA was reverse transcribed into cDNA using the Transcriptor First Strand cDNA synthesis kit (Roche, Indianapolis, IN, USA) and subjected to quantitative PCR using the SYBR-Green Supermix (Roche, Indianapolis, IN, USA) with the ABI Stepone Plus real-time PCR detection system (Applied Biosystems, Foster City, CA, USA). Using the 2^−ΔΔCT^ method [10.1006/meth.2001.1262], relative expression (fold change) of forkhead box O1 (FoxO1) (5′-AGCTCAAACGCTAGCACCAT-3′ and 5′-GGTGGATACACCAGGGAATG-3′), glucagon (5′-GCCGAGCAAGGCGAGACT-3′and 5′-CATGTCTGCGCCCAAGTTC-3′), insulin (5′-CCTGCCCAGGCTTTTGTCA-3′ and 5′-GGTGCAGCACTGATCCACAATG-3′), X-box binding protein 1 (XBP1) (5′-GGTCTCAGAGGCAGAGTCCAAG-3′ and 5′-AGAGGCAACAGCGTCAGAATCC-3′), Hspa5(BIP) (5′-GAGGACAAGAAGGAGGATG-3′ and 5′-TTGGACGTGAGTTGGTTC-3′), IL6 (5′-GTCAACTCCATCTGCCCTTC-3′ and 5′-TGTGGGTGGTATCCTCTGTG-3′), IL1β (5′-GCCAACAAGTGGTATTCTCCA-3′ and 5′-TGCCGTCTTTCATCACACAG-3′), HIFα (5′-TGGATGGCTTTGTTATGGTG-3′ and 5′-TGGTCACATGGATGGGTAAA-3′), and GAPDH (5′-TTAAGGGCATCCTGGGCTACACT-3′ and 5′-TTACTCCTTGGAGGCCATGTAGG-3′) was normalized to β-actin (5′-GTCGTACCACTGGCATTGTG-3′ and 5′-CTCTCAGCTGTGGTGGTGAA-3′) as the most suitable reference gene (expression level unaffected by the experimental treatment) and relative to the sham group as the calibrator. A no-template control reaction was included for each gene examined. All the primers were generated by Beijing Genomics Institute (Beijing, China).

### Western blotting

Western blots were developed as described previously^16^. The pancreatic tissue extracts for western blotting were prepared from four to six rats for each condition on experimental day P38. Thirty or forty micrograms of proteins were loaded into each well and fractionated on 5–10% precast SDS-PAGE gels (Byeotime, Shanghai, China) and then transferred to a PVDF membrane (Merck Millipore, Darmstadt, Germany). Membranes were probed using antibodies to FoxO1 (1:2500, 18592-1AP, Proteintech, Chicago, IL, USA), SOD2 (1:6000, 66474-1lg, Proteintech, Chicago, IL, USA), NOX4 (1:600, 14347-1-AP, Proteintech, Chicago, IL, USA), and β-actin (1:2000, AM10218-400, Abgent) as the primary antibodies. Goat anti-mouse IgG or anti-rabbit IgG (Boster, Wuhan, China) conjugated to HRP was used as the secondary antibody. Protein detection was accomplished using an enhanced BCA protein assay kit (Byeotime, Shanghai, China).

### Immunohistochemistry

On experimental day P38, pancreas samples were fixed in 2% w/v paraformaldehyde for 2 h and processed for immunohistochemistry as described previously^17^. After pre-blocking, pancreas samples were stained with primary antibodies in 5% v/v goat serum. A list of antibodies used in these studies is provided in Table 2. All secondary antibodies (Alexa Fluor 488 or 594 goat anti-mouse or anti-rabbit) were from Molecular Probes, Waltham, MA, USA. DAPI was used to stain nuclei. To show the outlines of acinar cells, we used Alexa Fluor 488 conjugated Wheat Germ Agglutinin (1:200) as counterstaining (W11261, Thermo Fisher, Waltham, MA, USA). Whole-sample images were scanned and assembled on a Leica fluorescence microscope (Leica DM6000B) using Leica LAS X software. Nikon A1R confocal microscope (Nikon, Japan) was applied to generate 3D rendered videos. For quantitative immunohistochemistry, the relative protein expression content/area determined from 160–170 fields (40×) of pancreas from each rat was quantified using ImageJ software. Immunoreactivity in each tissue section was normalized relative to the total area measured for each section. The tissue or cell immune-staining densities and their ratio were quantified by two independent operators in a blinded manner.

**Table 2:** A list of antibodies used in the studies

Antibody	Catalog no.	Source	Dilution
Primary antibody			
Mouse anti-superoxide dismutase 2 (SOD2) antibody	66474-1-Ig	ProteinTech, Chicago, IL, USA	1:300
Rabbit anti-forkhead box O1 (FOXO1) antibody	18592-1-AP	ProteinTech, Chicago, IL, USA	1:100
Rabbit anti-thioredoxin interacting protein (TXNIP) antibody	bs-3897R	Bioss, Beijing, China	1:50
Rabbit anti-neurogenin-3 antibody	bs-0922R	Bioss, Beijing, China	1:50
Mouse anti-insulin antibody	66198-1-Ig	ProteinTech, Chicago, IL, USA	1:1500
Rabbit anti-MAF bZIP transcription factor A (Mafa) antibody	Bs-0924R	Bioss, Beijing, China	1:25
Rabbit anti-pancreatic and duodenal homeobox 1 (PDX1) antibody	bs-0923R	Bioss, Beijing, China	1:25
Rabbit anti-glucagon antibody	PB0742	Boster, Wuhan, China	1:200
Rabbit anti-NOX4	14347-1-AP	ProteinTech, Chicago, IL, USA	1:100
Secondary antibody			
Alexa Fluor 488 goat anti-mouse IgG (H + L) antibody	A11001	ThermoFisher, Waltham, MA, USA	1:400
Alexa Fluor 488 goat anti-rabbit IgG (H + L) antibody	A11008	ThermoFisher, Waltham, MA, USA	1:400
Alexa Fluor 594 goat anti-rabbit IgG (H + L) antibody	A11012	ThermoFisher, Waltham, MA, USA	1:400
Alexa Fluor 594 goat anti-mouse IgG (H + L) antibody	A11005	ThermoFisher, Waltham, MA, USA	1:400

### TUNEL assay

On experimental day P38, pancreas samples were fixed in 2% w/v paraformaldehyde for 2 h and processed for TUNEL assay, which was performed using the One Step TUNEL Apoptosis Assay Kit (Beyotime, Shanghai, China) according to the manufacturer’s instructions. Slides were also counterstained with mouse anti-insulin primary antibody (1:1000, 4 °C overnight) and goat anti-mouse secondary antibody conjugated with Alex Fluor 594 (1:400, 1 h at room temperature). The TUNEL-positive cells with green fluorescence were detected under a fluorescent microscope (Leica DM6000B).

### Statistical analysis

Results are presented as the mean ± SEM. Statistical significance was determined by Student’s *t*-test or using analysis of variance (ANOVA) with Tukey tests for post hoc comparisons at a 1% or 5% significance level of difference. The relationships between variables were determined by linear regression analysis. GraphPad Prism 6.0 software (La Jolla, CA, USA) was used for statistical analyses.

## Results

### GSH prevented long-term oscillating glucose (LOsG) intake-induced prediabetes in rats

**Fig. 1:**
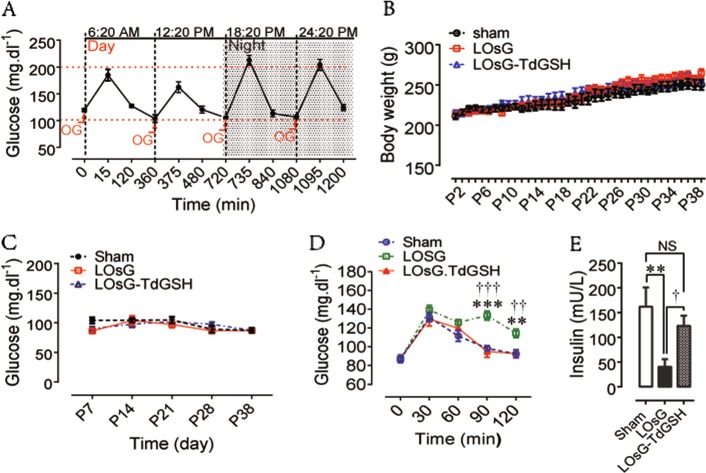
The role of OsG in metabolic homeostasis. Glycemic variation during 24 h (**a**), total body weight (**b**) and fasting blood glucose levels were measured as indicated. Day (**c**), OGTT (**d**), and fasting blood insulin (**e**) measurements were performed on P38. Data are shown as mean ± SEM (*n* = 6–10). **p < 0.01, ***p < 0.001 LOsG versus sham group; ^†^*p* < 0.05, ^††^*p* < 0.01, ^†††^*p* < 0.001 LOsG versus LOsG.GSH. NS, not significant

To mimic human food-intake habits in rodents, we orally (by gavage) gave female rats who were having regular rat chaw an extra 6 g/kg of d-glucose every 6 h per day for 38 days (P38), which resulted in an extra 1.6 times of sugar than what they usually have. During the daily OsG treatment cycle, plasma glucose was oscillating within the physiological range (between ~100–200 mg/dl, Fig. 1a).

LOsG did not obviously alter the body weight gaining, FBG levels (Fig. 1b, c), and plasma lipid profile (Table 3). However, LOsG impaired glucose tolerances (GT), as revealed by OGTT and decreased fast plasma insulin level (Fig. 1d, e), indicating that LOsG-impaired β-cell function and GT.

**Table 3:** Blood biochemical profiles

Group	*n*	TRIG (mmol/l)	CHOL (mmol/l)	HDL (mmol/l)	LDL (mmol/l)	LPS (mmol/l)
Sham	7	1.22 ± 0.75	1.17 ± 0.17	0.24 ± 0.04	0.07 ± 0.02	5.83 ± 1.76
LosG	6	0.49 ± 0.18	1.25 ± 0.19	0.26 ± 0.05	0.08 ± 0.02	3.85 ± 0.24
LosG.TdGSH	6	0.92 ± 0.55	1.20 ± 0.07	0.20 ± 0.03	0.17 ± 0.03	4.87 ± 0.30

*TRIG* triglyceride, *CHOL* cholesterol, *HDL* high density lipoprotein *LDL,* low density lipoprotein, *LPS* lipase

We have previously shown that GSH (50 mg/kg/6 h) totally blocks OsG/oxidative stress-induced disarrangement of partitions of circulating immune cells and neutrophil/lymphocyte ratio^17^. Theoretically, OsG/ROS stresses on β-cell should be relieved by a dynamic and timely administration of GSH (TdGSH); we therefore simultaneously gave animals GSH (50 mg/kg/6 h, subcutaneous injection) when they were receiving LOsG. Here we demonstrated that TdGSH eliminated the detrimental effects of LOsG on GT (Fig. 1d) and kept the fast plasma insulin level at normal levels (Fig. 1e), and thus could prevent the glucotoxicity-induced occurrence of prediabetes.

### TdGSH erased LOsG-induced cell metabolic memory (MM) in ROS homeostasis

Glucose/ROS pathways promote cellular adaptive processes, which can alleviate glucose-dependent ROS stress-induced detrimental effects on cell survival and subsequently allows for the development of MM^18^, which exists in diabetic patients. The organ damages following hyperglycemia/ROS stress can be hindered by initiating good glycemic control very early, but is not easily reversed if poorly handled over a longer time period^19^. To test whether LOsG can induce MM, we used WBC ROS accumulation as an index. On P38, blood cells from rats with 12 h-fasting were collected before and after 1 h of oral 2 g/kg glucose challenge. The WBCs were separated and the glycemic level and cell ROS accumulations were determined.

**Fig. 2:**
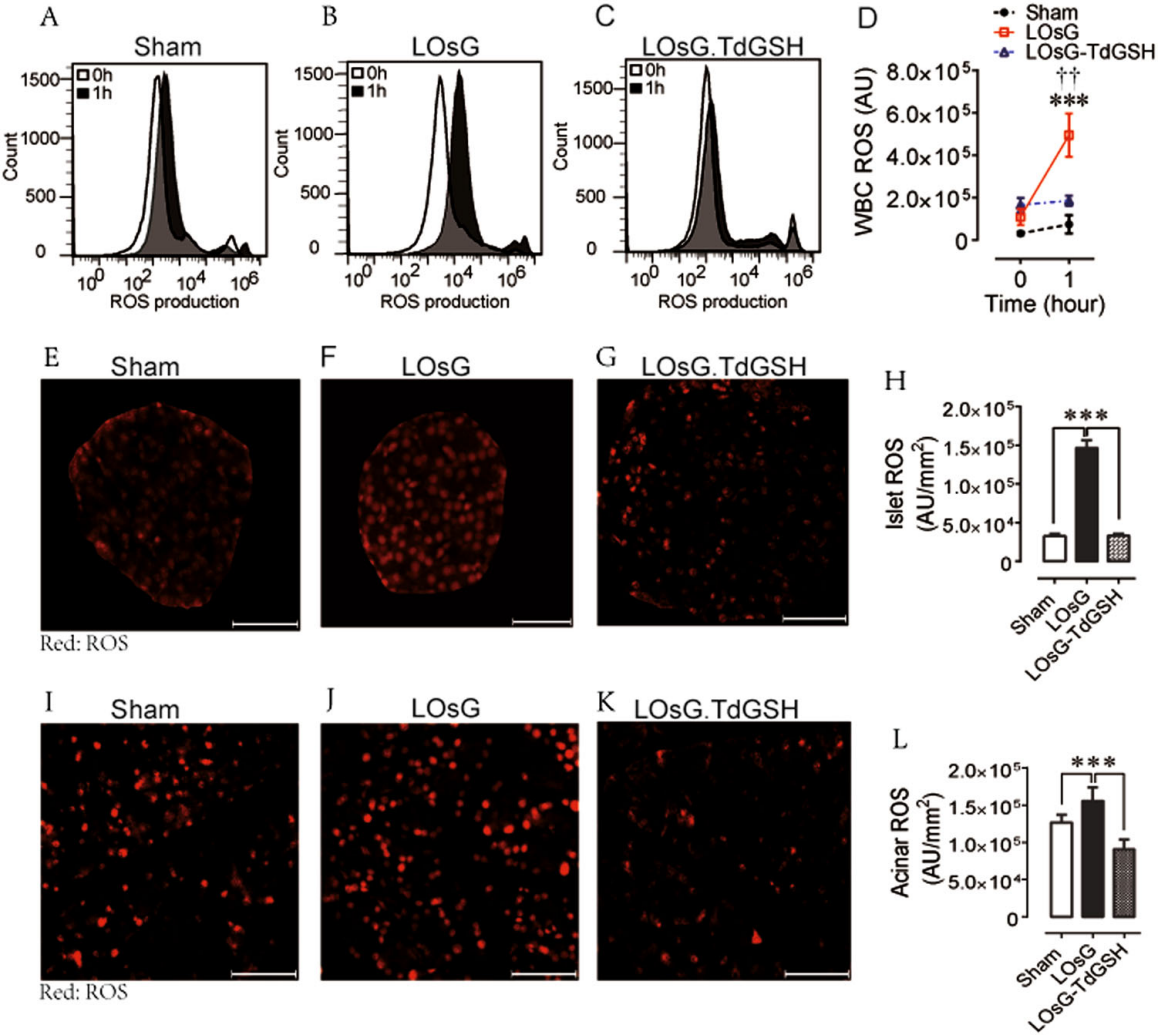
TdGSH prevents LOsG administration-induced alterations of ROS homeostasis. WBC ROS measurements (**a**–**d**) were performed at before zero hour (0 h) and one hour (1 h) after gavage of glucose (2 g/kg body weight) on P38. **a**–**c** Representative examples of flow cytometric analysis of WBC ROS in 3 groups. **d** Quantification of WBC ROS (***p < 0.001 LOsG versus sham; ^††^*p* < 0.01 LOsG versus LOsG.GSH). Examples of **e**–**g** islet and **i**–**k** acinar cell ROS staining in three groups (*n* = 6/group). **h**, **l** Quantification of ROS-reactive stains (red, presented as arbitrary unit/mm^2^) in islet and acinar tissue, respectively. Data are shown as mean ± SEM (*n* = 6–10; ***p < 0.001 represents both LOsG versus sham or LOsG.TdGSH). White photo bar is 50 μm

In spite of the fact that glycemic levels in all circumstances were similar among sham, LOsG, and LOsG.TdGSH-treated groups (Fig. S1A), the cell ROS dichlorofluorescein (DCF) assay in combination with flow cytometry analysis demonstrated that WBC ROS accumulations during 1 h of 2 g/kg glucose challenge in the LOsG group were significantly higher than in the sham group (Fig. 2a–d). In such circumstances, the driving force for the different cell ROS accumulating responses could be mainly contributing to the effects of MM induced by pre-LOsG treatment. Indeed, there was a positive correlation between glycemic level and WBC ROS accumulation only in LOsG-treated animals (*r* = 0.61, *n* = 14, *p* < 0.05; Fig. S1C) but not in other groups (Fig. S1B and D). This is consistent with a previous observation that T2DM patients exhibited greater blood mononuclear cell ROS accumulation compared to healthy controls^13^. Interestingly, glucose-stimulated magnitude of ROS accumulation in LOsG.TdGSH animals was similar to that of sham rats (Fig. 2a–d), suggesting that TdGSH totally erased the LOsG-produced MM in ROS accumulating responses in WBC. We further investigated the pancreatic cell ROS accumulation. In physiological condition, pancreatic acinar cell basal ROS concentration of sham rats was about 3.9-fold higher than that in β-cell. However, LOsG produced only 1.2-fold ROS increase in acinar cell (Fig. 2i, j), but 4.5-fold increase of ROS production in β-cell (Fig. 2e, f), indicating that β-cell has weak capability to handle ROS accumulation.

### TdGSH prevented LOsG–induced inhibition of pancreatic SOD-2 expression

**Fig. 3:**
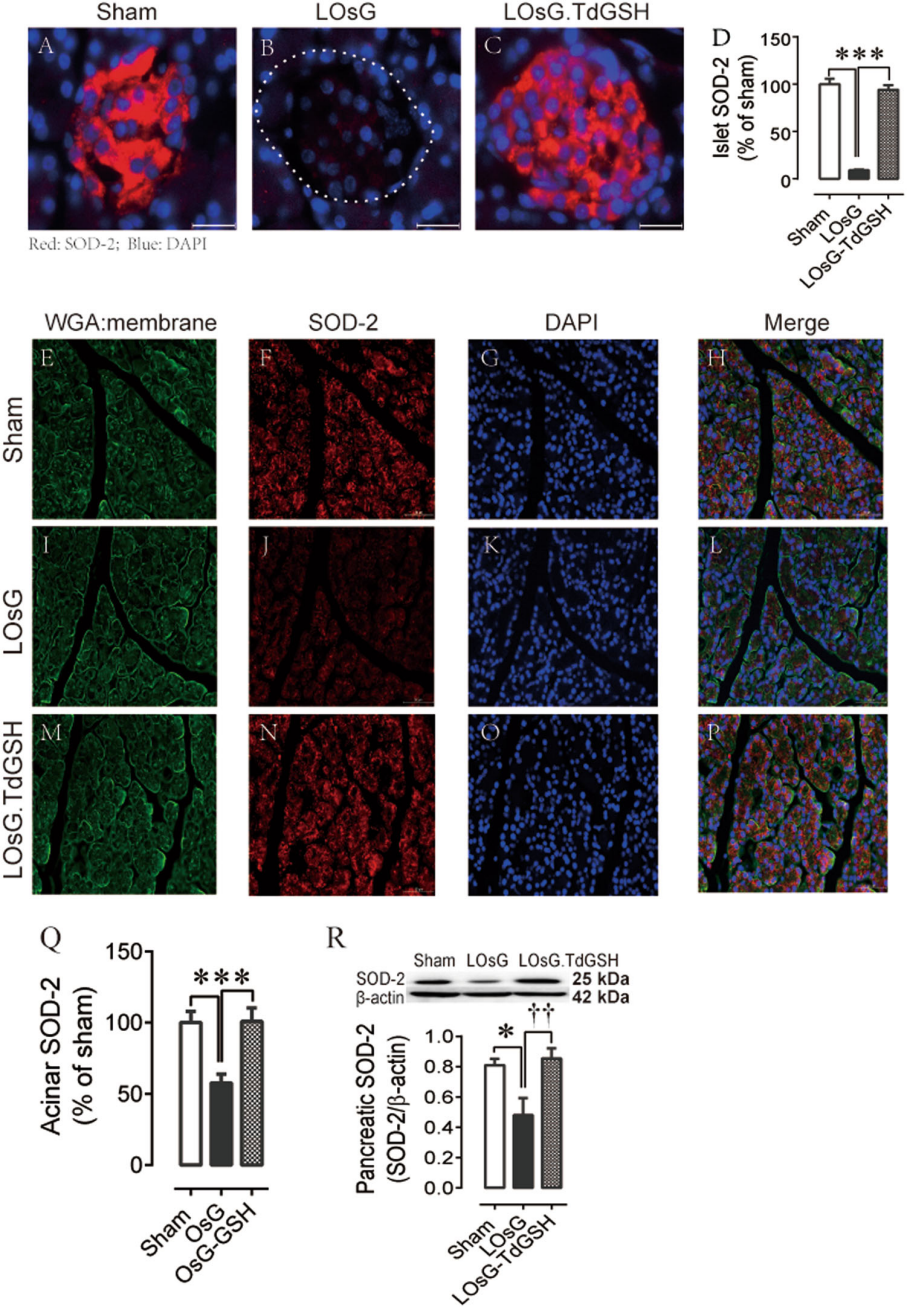
TdGSH prevents LOsG-induced inhibition of SOD-2 expression. **a**–**c** Examples of islet SOD-2 staining; **d** quantification of the islet SOD-2 immunoreactive area (red). **e**–**p** Examples of acinar SOD-2 staining; **q** quantification of the acinar SOD-2 immunoreactive area (red, *n* = 53–149 islets/group). ***p < 0.001 represents both LOsG versus sham or LOsG.TdGSH. **r** Western blot data of pancreatic SOD-2 (*n* = 4/group, *p < 0.05, ^††^*p* < 0.01). Data are shown as mean ± SEM. White photo bar is 50 μm

It is reported that the pancreatic β-cell has very low intrinsic levels of antioxidant proteins and activities and thus is very vulnerable to ROS^7,8^.We examined the antioxidant enzyme mitochondrial SOD-2 (SOD-2) expressions in pancreas and found that the relative SOD-2 immunoreactive content was 19.5-fold higher in islet than in acinar cells (Fig. 3a–f). However, islet cells were poor in handling the glucose/ROS stress. LOsG almost totally diminished SOD-2 expression in β-cells (Fig. 3b–d). Antioxidant TdGSH treatment fully prevented LOsG-induced ROS accumulation and SOD-2 diminution in β-cells (Fig. 3c, d). In contrast, although the expression of acinar cell SOD-2 was also inhibited by LOsG treatment, they still had 58% SOD-2 expression of sham (Fig. 3e–q). This is consistent with the results derived from pancreatic western blot analysis that 59% SOD-2 expression of sham remained in LOsG-treated pancreas (Fig. 3r).

In addition, double immunostaining with islet SOD-2 and insulin demonstrated that there was a strong positive correlation (*r* = 0.66, *n* = 133, *p* < 0.001) between islet SOD-2 and insulin expression (Fig. S2A–D), suggesting that SOD-2 is a critical enzyme to mitigate ROS accumulation and maintain β-cell function. We also determined the pancreatic NOX4 (NADPH oxidase 4), which expresses in rat pancreatic islets^20^, and found that LOsG did not significantly alter NOX4 protein expression (Fig. S3), indicating that, relative to SOD-2, NOX-4 played a lesser role with regard to LOsG/ROS stress effects on β-cell.

### TdGSH prevented LOsG-induced β-cell failure

**Fig. 4:**
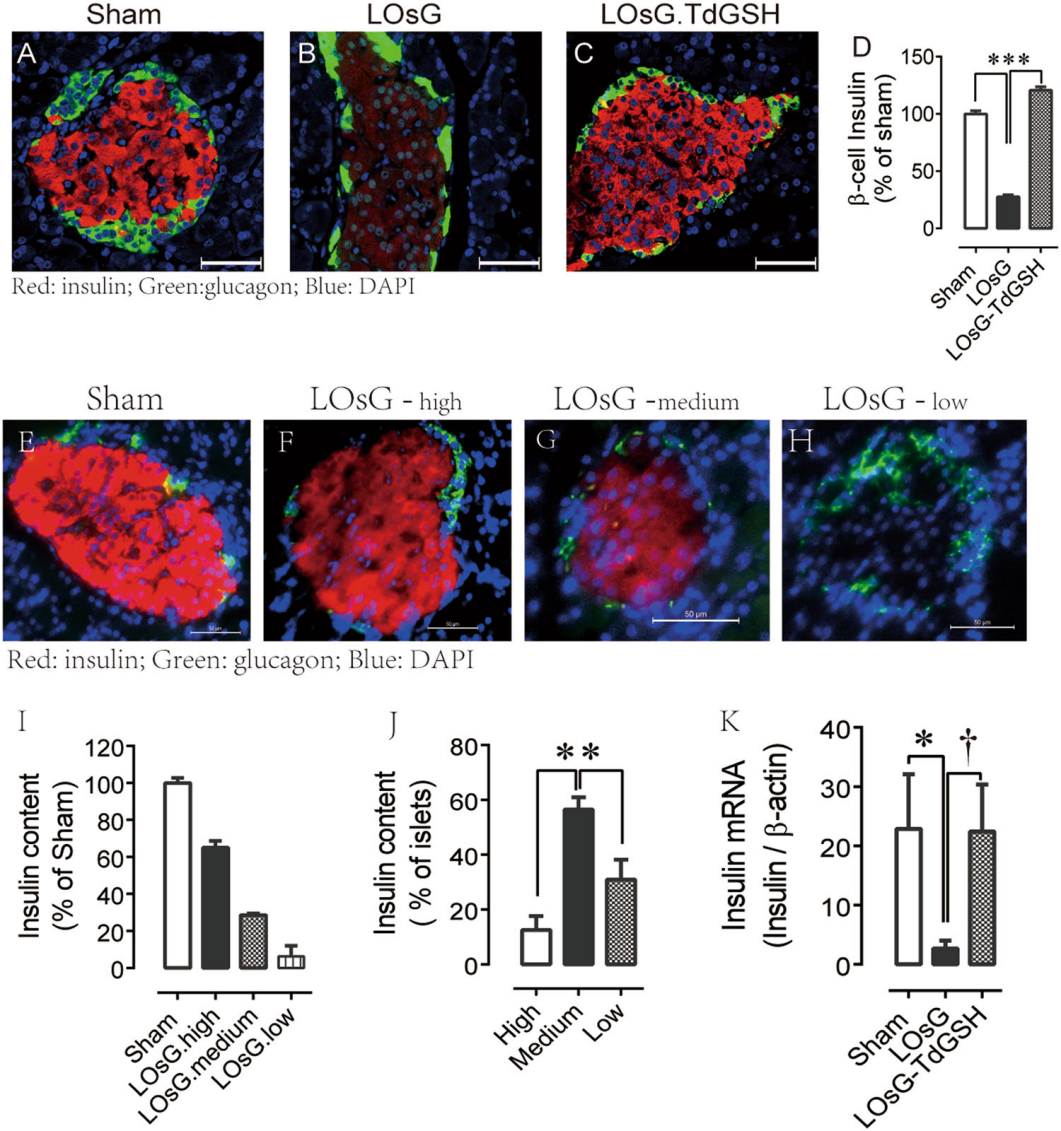
TdGSH prevents LOsG-induced inhibition of insulin expression. **a**–**c** Examples of islet insulin content; **d** quantification of insulin immunoreactivities (red). The data were plotted as percentage of sham group (*n* = 117–167 islets/group; ***p < 0.001 represents both LOsG versus sham or LOsG.TdGSH). **e**–**h** Examples of islet insulin content of sham, LOsG-high, medium, and low insulin staining. **i** Quantification of islet insulin contents of LOsG-high, medium, and low classified groups relative to that of sham. **j** Distributions (%) of LOsG-high, -medium, and -low insulin content among pancreatic islets. **k** Quantification of insulin mRNA on P38 (*n* = 4–6/group, * or ^†^*p* < 0.05). Data are shown as mean ± SEM. White photo bar is 50 μm

Although associations between the glucose/ROS toxicity and β-cell insulin gene expression have been established in vitro^21^, no long-term toxic effects of OsG in vivo have yet been reported. Here we demonstrated that LOsG in normal animals significantly decreased pancreatic islet insulin content (Fig. 4a–d) and insulin mRNA expression (Fig. 4k). Again, TdGSH fully prevented LOsG-induced detrimental effects on insulin production (Fig. 4a–k). Because GSH is a strong endogenous antioxidant, these data indicate that chronic oxidative stress is the major mechanism of glucotoxicity in the pancreatic β-cell.

Interestingly, immunohistochemistry analysis revealed a heterogeneously functional population of islets in LOsG-treated pancreas. Depending on the islet insulin immunoreactivities in the LOsG group, islets can be classified into three populations: 12.6 ± 5.0% of high (i.e., insulin content is 65 ± 4% of average sham islet), 56.5 ± 4.4% of medium (insulin content is 29 ± 1% of sham), and 30.9 ± 7.3% of low (insulin content is 6 ± 1% of sham, almost undetectable) insulin content islet (Fig. 4e–j). These findings are consistent with previous observations that large inter-islet insulin expression variations exist even within the same pancreas of FoxO1 transcriptional factor knockout animal^22^.This suggests that 31% of the islets in LOsG-treated pancreas have lost their β-cell identity, and β-cells may be dedifferentiated and/or transdifferentiated into other cell types. The LOsG decreased β-cell insulin content might be mainly at the transcriptional level, as the insulin mRNA was significantly lower in the LOsG group than in sham animals (Fig. 4k). Again, TdGSH fully blocked the LOsG-induced decreases of insulin mRNA and protein expression (Fig. 4d–k).

### LOsG produced very limited β-cell loss by apoptosis

In common with other cell types, an increased vulnerability to apoptosis is considered as the main cause of β-cell loss^23^. In light of the plausible proapoptotic role of ROS, we performed apoptotic cells analysis by TUNEL assays, but saw that LOsG did not significantly increase apoptotic cell numbers in islets in relative to the sham treatment (Fig. S4A–D; *p* > 0.05). As there was no differences of islet area nor islet density between groups (Fig. S5A and S5B), the reasons for β-cell functional failure in LOsG group could be hardly explained by the limited extra loss of β-cells on LOsG treatment day P38.

### TdGSH prevented LOsG-induced functional failure of dedifferentiated β-cell via the FoxO1/TXNIP pathway

**Fig. 5:**
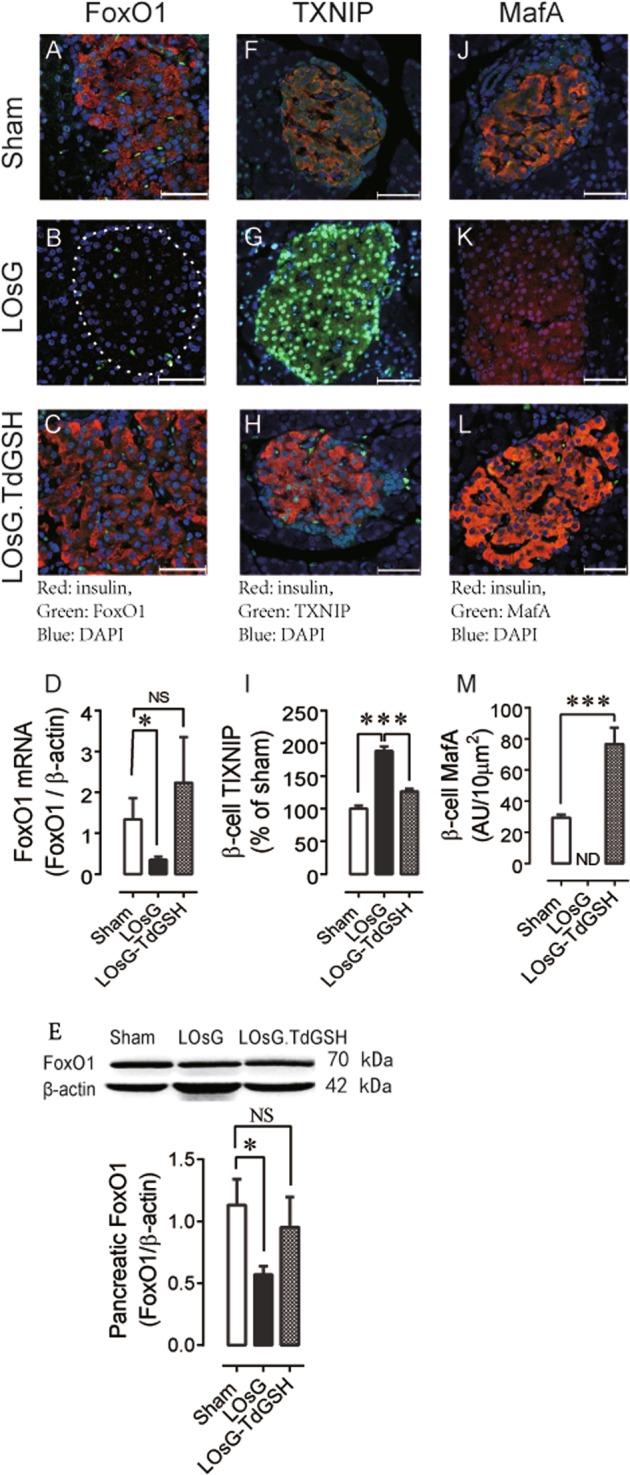
TdGSH prevents LOsG-induced alterations of FoxO1, TXNIP, and MafA expressions. **a**–**c** Examples of islet FoxO1 staining (green) in sham, LOsG, and LOsG.TdGSH groups (*n* = 6/group); **d** FoxO1 mRNA quantification from the same pancreatic tissues. **e** Quantification of pancreatic FoxO1 by western blot (*n* = 4/group, ***p < 0.001). **f**–**h** Examples of islet TXNIP staining; **i** Quantification of β-cell nuclear TXNIP immunoreactivities (green, *n* = 96–100 β-cells/group, plotted as percentage of sham group). **j**–**l** Examples of islet MafA expression; **m** Quantification of MafA immunoreactivities (green, *n* = 90–93 β-cells/group, plotted as AU/ 10 μm^2^). ND, not detectable. Data are shown as mean ± SEM. ***p < 0.001 represents both LOsG versus sham or LOsG.TdGSH. NS, not significant. White photo bar is 50 μm

Pancreatic β-cell dedifferentiation is one of the mechanisms resulting in diabetic β-cell failure. Given the FoxO1 ablation induced β-cell dedifferentiation and caused hyperglycemia under physiologic stress^22^, we determined whether LOsG/ROS stresses decrease FoxO1 expression and thus induce β-cell dedifferentiation. Indeed, compared to the sham group, LOsG significantly decreased both FoxO1 mRNA and protein expression, as demonstrated by immunohistochemistry analysis (Fig. 5a–c; Fig. S6), qRT-PCR (Fig. 5d), and western blot (Fig. 5e) on P38. TdGSH prevented LOsG-induced FoxO1 loss. Interestingly, double immunostaining with FoxO1 and insulin demonstrated that there was a positive correlation between FoxO1 and insulin (Fig. S7), indicating that, in addition to maintaining β-cell identity, FoxO1 might directly regulate insulin expression.

As known, FoxO1 inhibits expression of thioredoxin interacting protein (TXNIP)^24^. TXNIP downregulates V-maf musculoaponeurotic fibrosarcoma oncogene homolog A (MafA), a transcription factor to drive insulin expression, and thus negatively regulates insulin expression^25^. We therefore examined TXNIP and MafA expression in β-cell. In relative to the β-cell of the sham group, LOsG resulted in 3.3-fold increase of nuclear TXNIP (Fig. 5f–i), which was associated with 4.9-fold decrease of nuclear MafA expression (Fig. 5j–m). TdGSH again fully blocked LOsG-induced increase/decrease of TXNIP/MafA expression, respectively. There was a negative correlation between TXNIP and insulin (Fig. S8) and a positive correlation between MafA and insulin expression (Fig. S9). Taken together, these results show that LOsG downregulates FoxO1, which releases its suppression effects on TXNIP. The upregulated TXNIP downregulates MafA and thus inhibits insulin expression in LOsG-treated β-cell.

**Fig. 6:**
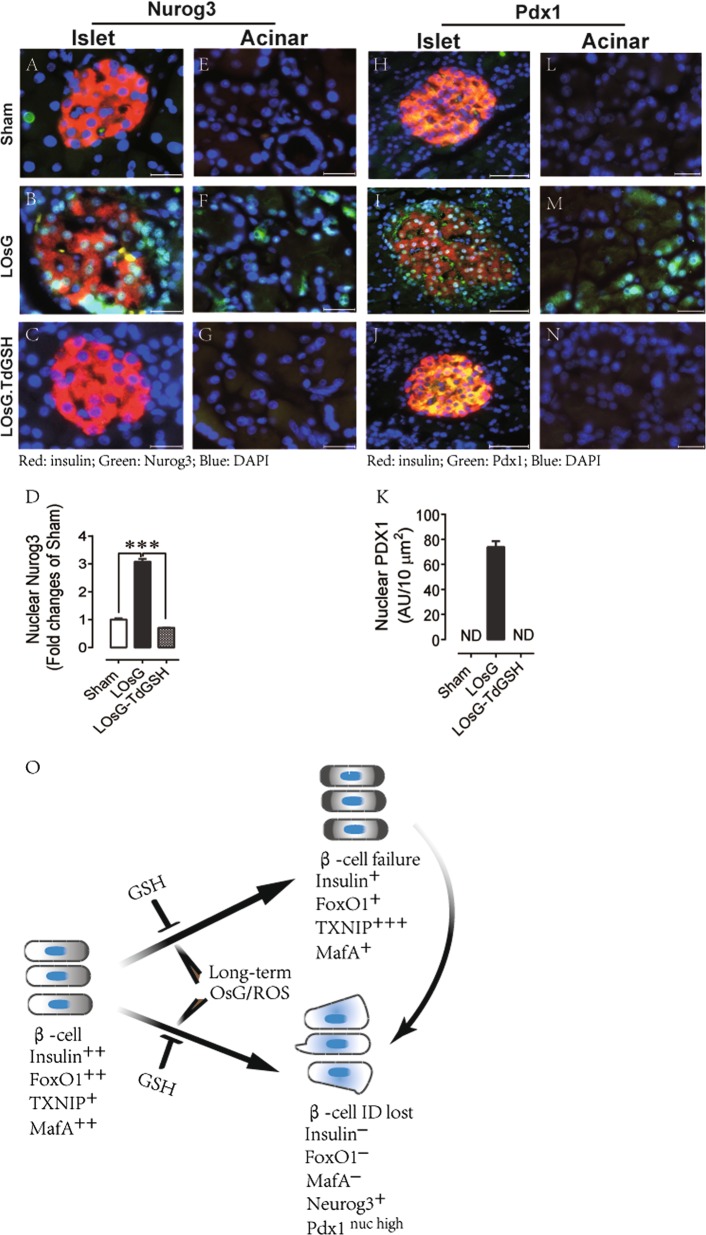
TdGSH prevents LOsG-induced β-cell dedifferentiation. Examples of **a**–**c** islet and **e**–**g** acinar cell Nurog3 staining in sham, LOsG, and LOsG.TdGSH groups (*n* = 6/group); **d** is quantification of β-cell nuclear Nurog3 immunoreactivities (green, *n* = 93–100 cells/group; presented as fold changes of sham group) in islet. Examples of **h**–**j** islet and **l**–**n** acinar cell Pdx1 staining; **k** quantification of β-cell nuclear Pdx1 immunoreactivities (green, *n* = 95–99 cells/group; plotted as AU/ 10 μm^2^). Data are shown as mean ± SEM. ***p < 0.001 represents both LOsG versus sham or LOsG.TdGSH. ND, not detectable. White photo bar is 50 μm. **O** A proposed mechanism of β-cell failure. Healthy β-cells produce insulin^++^ and have appropriate expressions of FoxO1^++^, TXNIP^+^, and MafA^++^. Long-term extra intake of carbohydrate diet could cause supraphysiological blood oscillating glycemia and ROS stress. Under this stress, β-cells would transition to two major cell subtypes depending on their traits: (1) β-cell functional failure characterized by the fall of FoxO1 expression, which in turn causes the increase of TXNIP expression and subsequently decrease MafA and insulin expression, and (2) β-cells losing their identification, which is associated with reactivation of pancreatic endocrine progenitor gene Neurog3 and Pdx1 expression, and the total loss of their capabilities to produce insulin. Subtype (1) could further transition to subtype (2) of β-cell if the stress persists. GSH can prevent all detrimental effects caused by OsG on β-cells

We further examined the effects of LOsG on β-cell dedifferentiation. FoxO1-deficient β-cells lose their identity and revert to pancreatic endocrine progenitors characterized by expressing high levels of neurogenin-3 (Neurog3 ^high^), and no insulin and MafA expression^22^. Interestingly, on undergoing a LOsG/ROS stress, β-cells started to re-express fetal nuclear Neurog3 and to dramatically relocate pancreas/duodenum homeobox protein 1 (Pdx1) from the cytoplasm to the nucleus (Fig. 6a–k). This is consistent with previous findings that β-cell plasticity occurs in common forms of β-cell dysfunction, and is caused by the downregulation of FoxO1 that follows hyperglycemia-induced oxidative stress^26^. TdGSH totally blocked LOsG-induced β-cell dedifferentiation and prevented Neurog3 expression and relocation of nuclear Pdx1 immunoreactivities (Fig. 6d–k). In addition, some acinar cells also exhibited high levels of nuclear Neurog3 and Pdx1 under LOsG/ROS stress (Fig. 6f–m). During pancreatic development, Neurog3 and Pdx1 are expressed in pancreatic progenitor cells and controls cells differentiation. Acinar cells and endocrine islet cells are generated from the common progenitor after the duct cell lineage has already separated^27^. In the present studies, the re-expression of fetal genes in both β-cell and acinar cell suggests that LOsG/ROS stress drives pancreatic cells to undergo dedifferentiation and revert to their common progenitor-like stage.

### A subset of dedifferentiated β-cells were at early stage to convert into α-cell in LOsG-treated animals

As a subset of FoxO1-deficient β-cells adapts the α-cell fate, resulting in hyperglucagonemia^22^, we determined pancreatic glucagon mRNA in LOsG-treated β-cells, which were associated with low or absent FoxO1 expression. We found that there were no significant differences in glucagon mRNA expression between LOsG and other groups (Fig. S10C). However, we did occasionally see the β-cells located in the middle of a subset of islets appearing as a double-stained faint glucagon and low-insulin protein (Fig. S10D). This might be because the limited time of LOsG treatment resulted in a conversion of a subset of β-cells to α-cells at early stage, and others had not started to change yet.

### LOsG did not alter expressions of pancreatic ER stress, inflammation, and hypoxia-responsive genes

It has been suggested that endoplasmic reticulum (ER) stress, inflammation, and hypoxia are related to the underlying mechanism of β-cell dedifferentiation^28^. Thus, we determined ER-stress response genes Xbp1 and the ER chaperone heat shock 70 kDa protein 5 (HSPA5, previously known as immunoglobulin heavy chain binding protein [BIP]), inflammation-responsive genes IL6 and IL1β, and hypoxia-responsive genes hypoxia-inducible transcription factor 1α (HIF1α) and GAPDH. We found that LOsG did not alter the mRNA levels of ER-stress responsive genes Xbp1 and BIP, inflammation-responsive genes IL6 and IL1β, and hypoxia-response genes HIF1α and GAPDH compared with sham control mice (Fig. S11).

## Discussion

We provide, for the first time, direct evidence that only 38 days of extra oral glucose intakes four times a day can induce prediabetes. Instead of genetic knockout, functional knockout of FoxO1 occurs naturally when β-cells undergo LOsG/ROS stress. When stress persists, the β-cell gradually retreats and converts into a dedifferentiated pancreatic progenitor, which might be more resistant to high-glucose/ROS challenges^22,29^ and thus survives. Hyperglycemia has been shown to negatively regulate FoxO1 expression and paralleled loss of insulin content^22^. In this study, we demonstrate that long-term supraphysiological range of OsG/ROS stresses disrupts the FoxO1/TXNIP pathway and results in β-cell dedifferentiation, mass loss, and failure. This suggests that the extra carbohydrate intake in multiple times a day particularly damages β-cells.

It was only recognized in the late 1980s that desensitization of insulin secretion, which describes a state of decreased secretory responsiveness of β-cell to the glucose stimuli, is a pathophysiological phenomenon of β-cell function and not just an in vitro artifact^30^. There are two opposing views with regard to the underlying mechanism of this phenomenon. The first is that glucose-induced desensitization is due to the glucose insensibility of the β-cell; the second is that the long-term stimuli-increased secretory activity leads to a depletion of the releasable insulin of β-cells^31^. Here, we demonstrated that long-term oscillating glucose challenge in vivo resulted in desensitization of insulin secretion due to β-cell mass exhaustion, which was caused by β-cell dedifferentiation. At the early stage of prediabetes, the OGTT results demonstrated that the insulin exhaustion appeared in the second phase of insulin release. The impaired glucose tolerance in LOsG-treated animals became obvious at 90 and 120 min in OGTT. In addition, the fasting plasma insulin level was significantly lower in LOsG-treated animals than in sham rats. Our results are clinically relevant and consistent with clinical reports that the secretory capacity of β-cell was diminished in first-degree relatives of individuals with T2DM even before the onset of obesity and insulin resistance^32^.

Hyperglycemia and the ensuing glucotoxicity exert their deleterious effects via myriad mechanisms, including ROS stress, ER stress, inflammation, and hypoxia^28^. In LOsG-induced prediabetes, although pancreatic ROS stress remained high on LOsG treatment day 38, there were no alterations in mRNA levels of ER stress (XBP1 and BIP), inflammation (IL6 and IL1β), and hypoxia-responsive (HIF1α and GAPDH) genes between LOsG- and sham-treated groups. This is consistent with the previous findings that hypoxia-response genes were upregulated in the islets of diabetic, but not prediabetic, db/db mice in an inverse relationship with unfolded protein response gene expression^28,33^. In addition, administration of the antioxidant GSH can fully prevent all of the LOsG-induced detrimental events. Our data suggest that only an increase in oxidative stress resulting from LOsG is sufficient to induce pancreatic β-cell dedifferentiation.

Although clinical trials have so far failed to demonstrate relevant clinical benefits of antioxidant (such as vitamin C, vitamin E, and α-lipoic acid) to treat T2DM^34^, here we show that TdGSH can fully prevent LOsG/ROS-induced detrimental effects on β-cells. The practical application of antioxidant to prevent OsG/ROS stress-induced damages would be in the endogenous antioxidant form,GSH. The plausible main reason for β-cell protective effects of GSH differing from other antioxidant effects is that GSH has multiple functions, such as maintaining levels of reduced glutaredoxin and glutathione peroxidase^35^ and maintaining exogenous antioxidants such as vitamins C and E in their reduced (active) forms^36–38^. Alterations of GSH level can regulate redox changes to nuclear proteins necessary for the cell maturation^39^. The other possible reasons are that subcutaneous and timely administration of TdGSH helps in gaining sufficient antioxidant concentration in time to protect LOsG-induced β-cell damages. The clinical implication of our findings is to use TdGSH to prevent progressive loss of β-cell mass in diabetic patients, who have more glycaemic variabilities because of the vicious metabolic memory obtained from the disease, and to block the development of diabetic complications. We propose that long-term extra sugar intake multiple times a day can gradually sweep the β-cells away, and suggest that simultaneously taking glutathione, an antioxidant, in each meal could prevent blood glucose/ROS-induced detrimental effects on β-cell.

## Supplementary information

Supplemental material

Figure S1

Figure S2

Figure S3

Figure S4

Figure S5

Figure S6

Figure S7

Figure S8

Figure S9

Figure S10

Figure S11

Movie s1

Movie s2

Movie s3
