# Supplementary material for: Glutathione prevents chronic oscillating glucose intake-induced β-cell dedifferentiation and failure
Source: Cell Death Dis. 2019 Apr 11;10(4):321. doi: 10.1038/s41419-019-1552-y (PMC6459929; doi:10.1038/s41419-019-1552-y)
Supplement: Supplementary file 1 — Supplemental material [file 41419_2019_1552_MOESM1_ESM.docx]

Supplementary Materials for

**Glutathione prevents orally chronic oscillating glucose intakes induced β–cell dedifferentiation and failure**

**Authors:** Jitai Zhang^1†^, Hui An^1†^, Kaidi Ni^1†^, Bin Chen^2†^, Hui Li^1, 2^, Yanqin Li^1^,Guilian Sheng^1^, Chuanzan Zhou^1^, Mengzhen Xie^1^, Saijing Chen^1^, Tong Zhou^1,3^, Gaoxiong Yang^1^, Xiufang Chen^4^, Gaojun Wu^5*^, Shengwei Jin^6*^, Ming Li^1*^

*Correspondence to: Ming Li*, Email [mingli@wmu.edu.cn](mailto:mingli@wmu.edu.cn), Shengwei Jin*, Email [jinshengwei69@163.com](mailto:jinshengwei69@163.com) or Gaojun Wu*, Email [2855930357@qq.com](mailto:2855930357@qq.com)

† These authors contributed equally to this Work.

**This file includes:**

Figs. S1 to S11

Captions for Movies S1 to S3

**Other Supplementary Materials for this manuscript include the following:**

Movies S1 to S3

Fig. S1. A positive correlation between glycaemic level and WBC ROS content was only observed in LOsG treated animals. A. Plasma glucose levels in LOsG group did not differ from other groups at pre- and one hour post-2 g/Kg oral glucose challenge (n=6-7). B. No correlation between glycaemic level and WBC ROS in sham group. C. A positive correlation between glycaemic level and WBC ROS in LOsG treated animals.D. No correlation between glycaemic level and WBC ROS in LOsG.TdGSH group.

Fig. S2. TdGSH prevented LOsG induced decrease of SOD-2 and insulin protein expressions. A-C. Examples of SOD-2 (red) and insulin (green) immunostaining in sham (A), LOsG (B) and LOsG.TdGSH (C) treated islets. Color brown or yellow is the merge of red with green. White photo bar is 50 μm. D. There was a positive correlation between SOD-2 and insulin expressions. AU, arbitrary unit.

Fig. 3. LOsG did not significantly alter pancreatic NOX4 expression (n=3-5/group). Data are shown as mean ± SEM. NS, not significant.

Fig. S4. LOsG treatment did not significantly increase apoptotic cells in islet. In TUNEL assays, islet apoptotic cells was hardly observed in sham (A) and LOsG.TdGSH groups (C). B. An example of apoptotic cells in LOsG-treated islet. DNA is counterstained with DAPI. D. Quantification of apoptotic nuclei as detected by TUNEL assays in sham, LOsG and LOsG.TdGSH animals (n = 6 / group).There was no significant difference of % apoptotic cells/islet nuclei between groups. NS, not significant. White photo bar is 50 μm.

Fig. S5. LOsG treatment did not significantly alter islet area and density. A. Islet area (127 – 168 islet areas/592.0 – 690.5 mm^2^ in each group, n=6/group). B. Islet density (127 – 168 islets/592.0 – 690.5 mm^2^ in each group, n=6/group).

Fig. S6. Three D images show FoxO1 and insulin protein expressions and localizations in islet. A. In sham islet, FoxO1 (green) is localized at both cytoplasm and nuclei. DNA is counterstained with DAPI. Orange colour shows the mixture of green (FoxO1) and red (insulin). White colour shows the mixture of green (FoxO1) and blue (nuclear). B. FoxO1 and insulin expressions were dramatically diminished in LOsG treated islets. C. In LOsG.TdGSH islet, FoxO1 was maintained its expression as in sham group.

Fig. S7. There was a positive correlation between β-cell FoxO1 and insulin protein expressions.

Fig. S8. There was a negative correlation between β-cell TXNIP and insulin protein expressions.

Fig. S9. There was a positive correlation between β-cell MafA and insulin protein expressions.

Fig. S10. A subset of islets in LOsG treated animal dramatically decreased β-cell insulin expression and initiated the glucagon expression. A and B. Examples of islet glucagon and insulin expression in sham (A) and LOsG.TdGSH (B) groups. C. Pancreatic glucagon mRNA expressions in sham, LOsG and LOsG.GSH groups (n=4-6). There was no significantly difference of glucagon mRNA expression between LOsG with sham group (p>0.05). D. An example of islet glucagon and insulin expression in LOsG treated animal. DNA is counterstained with DAPI. White photo bar is 50 μm.

Fig. S11. LOsG did not alter pancreatic ER-stress response genes X-box binding protein 1 (Xbp1, A) and immunoglobulin heavy chain binding protein (BIP, B), inflammation responsive genes IL6 (C) and IL1β (D) and hypoxia-responsive genes HIF1α (E) and GAPDH (F) mRNA expressions (n=5-6 for all groups).

Fig. S1.


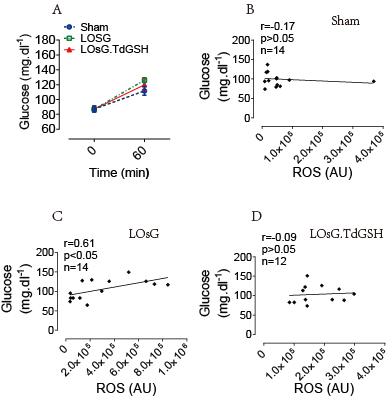


Fig. S2.


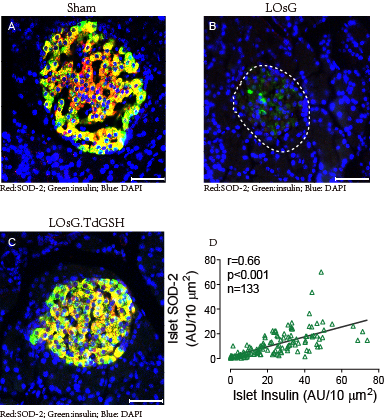


Fig. S3.


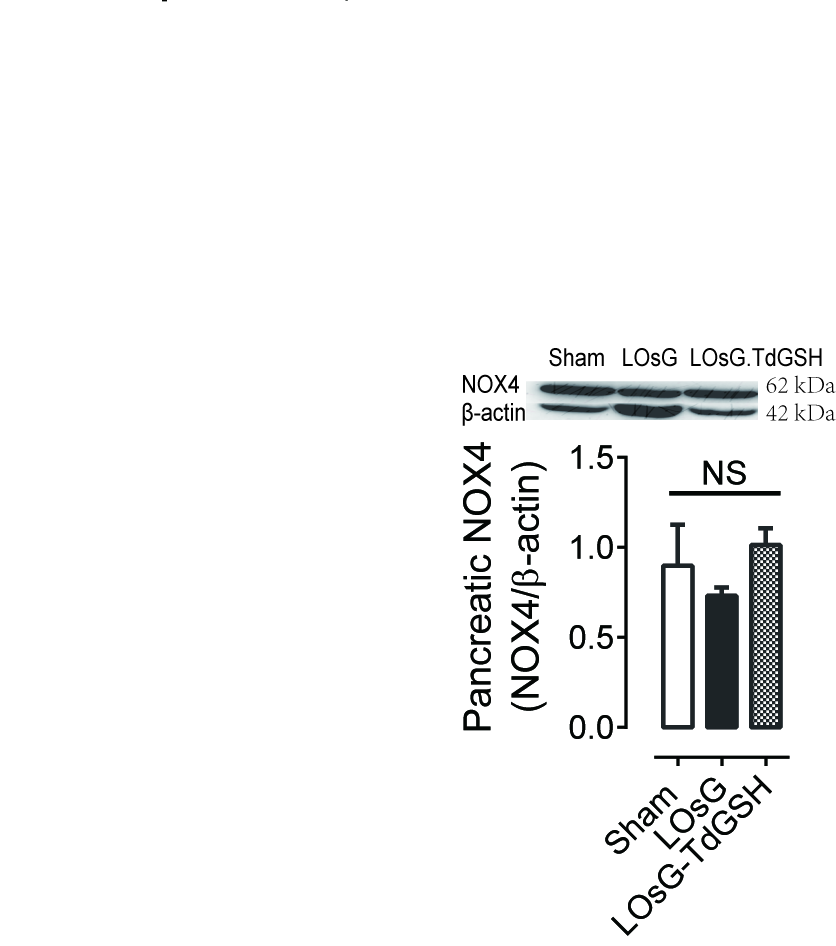


Fig. S4.


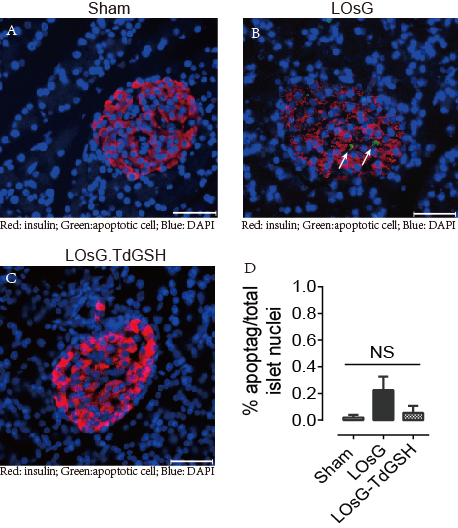


Fig. S5.


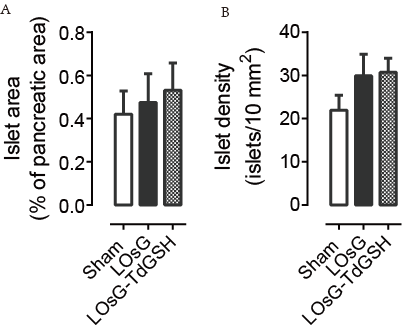


Fig. S6.


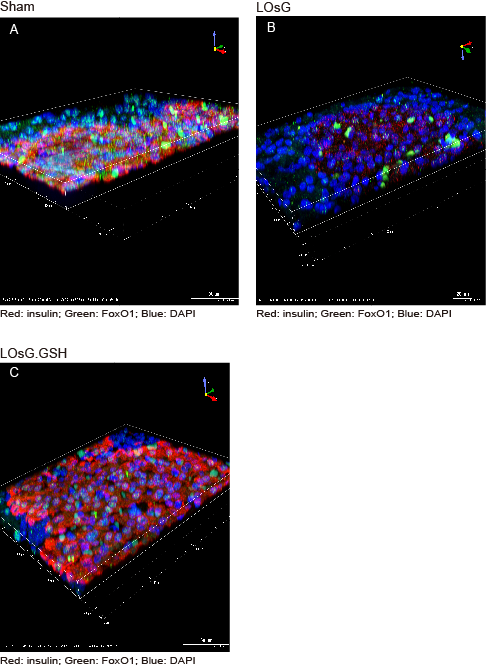


Fig. S7.


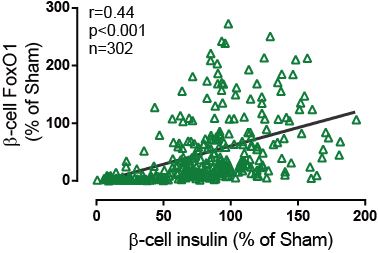


Fig. S8.


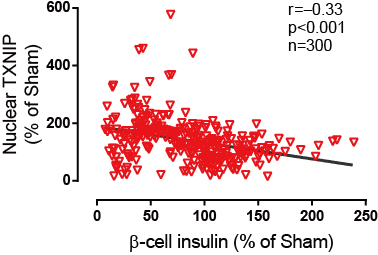


Fig. S9.


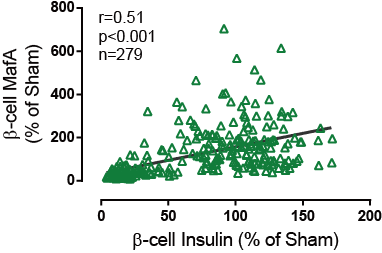


Fig. S10.


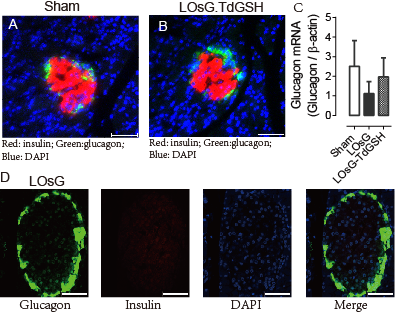


**Fig. S11**


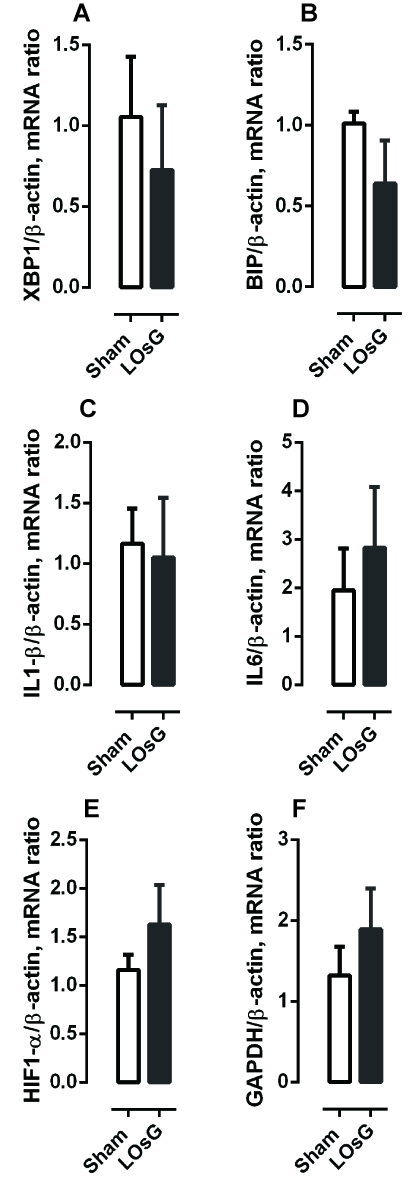


Movie S1. Islet FoxO1 (green) and insulin (red) immunoreactivities in sham group

Movie S2. Islet FoxO1 (green) and insulin (red) immunoreactivities in LOsG group

Movie S3. Islet FoxO1 (green) and insulin (red) immunoreactivities in LOsG.TdGSH group
