# Supplementary figures and images for: Glutathione prevents chronic oscillating glucose intake-induced β-cell dedifferentiation and failure
Source: Cell Death Dis. 2019 Apr 11;10(4):321. doi: 10.1038/s41419-019-1552-y (PMC6459929; doi:10.1038/s41419-019-1552-y)

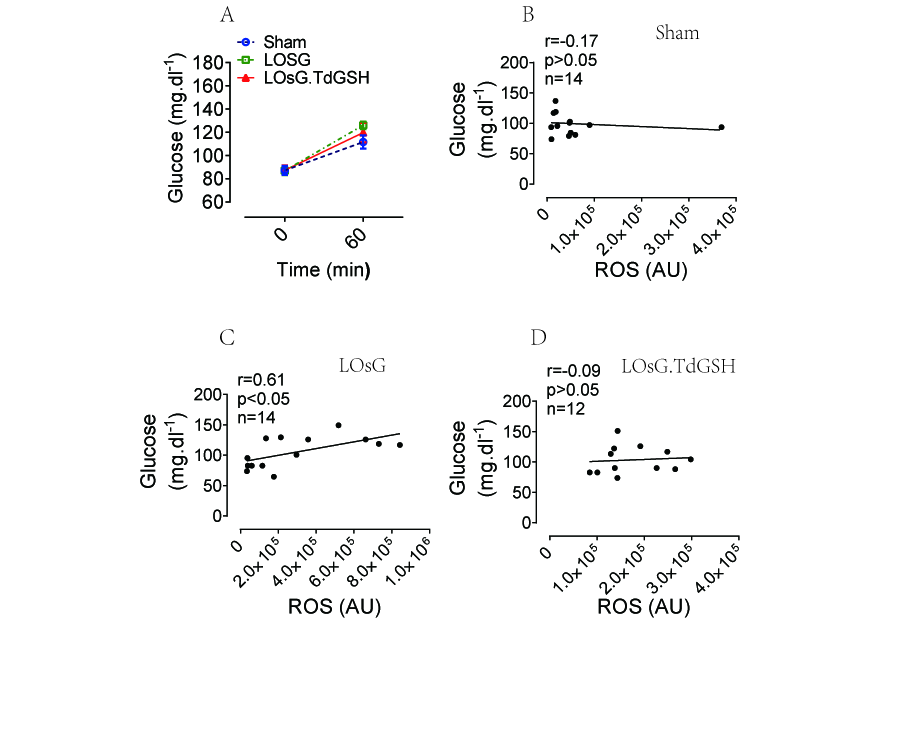

Supplement: Supplementary file 2 — Figure S1 [file 41419_2019_1552_MOESM2_ESM.tif]

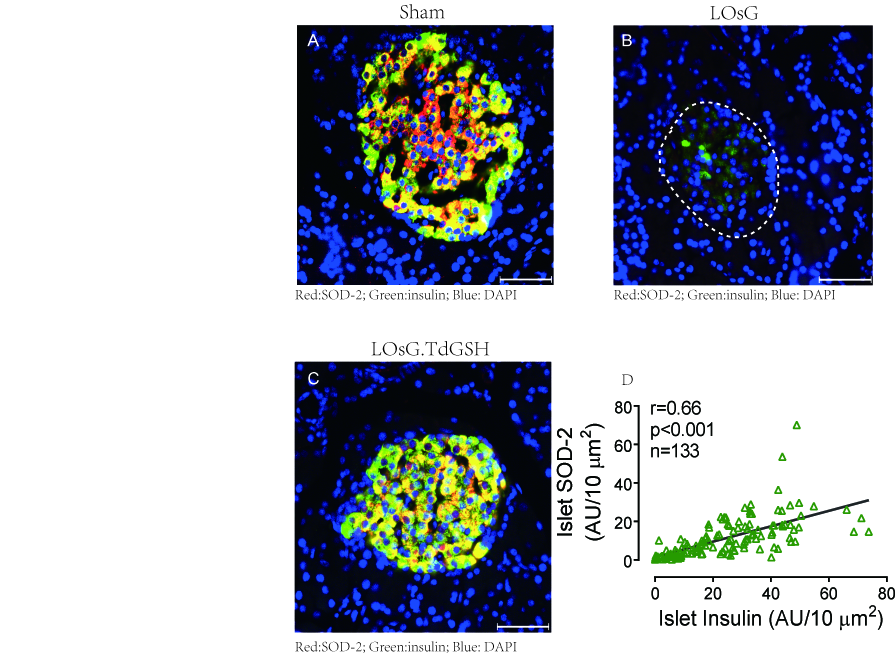

Supplement: Supplementary file 3 — Figure S2 [file 41419_2019_1552_MOESM3_ESM.tif]

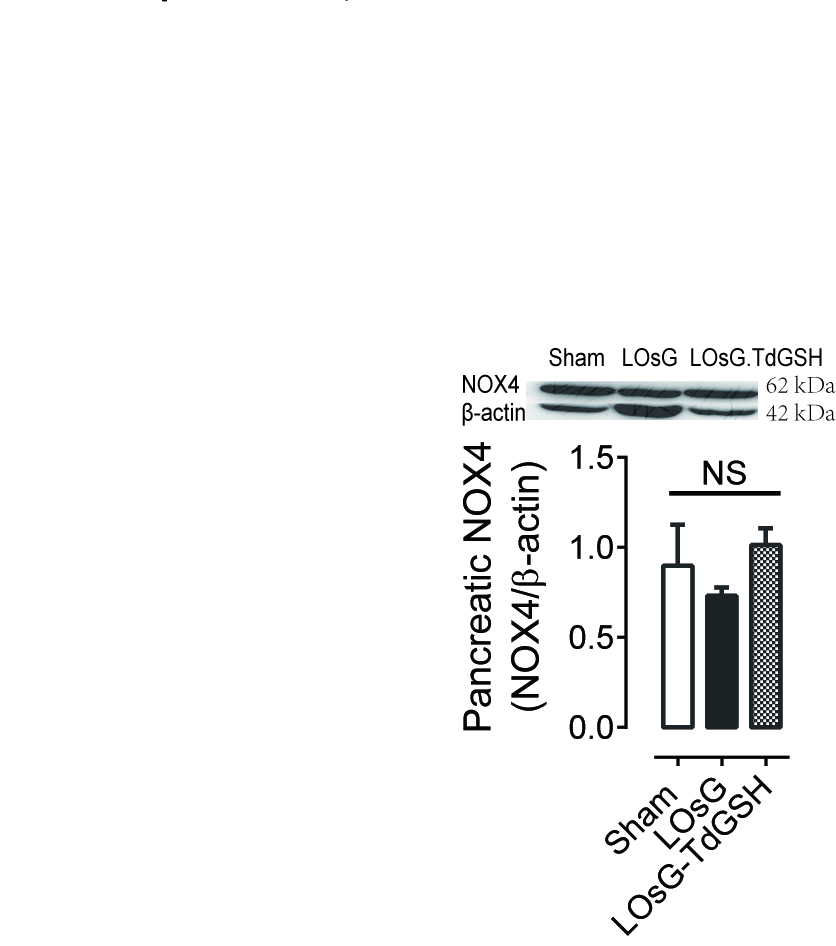

Supplement: Supplementary file 4 — Figure S3 [file 41419_2019_1552_MOESM4_ESM.tif]

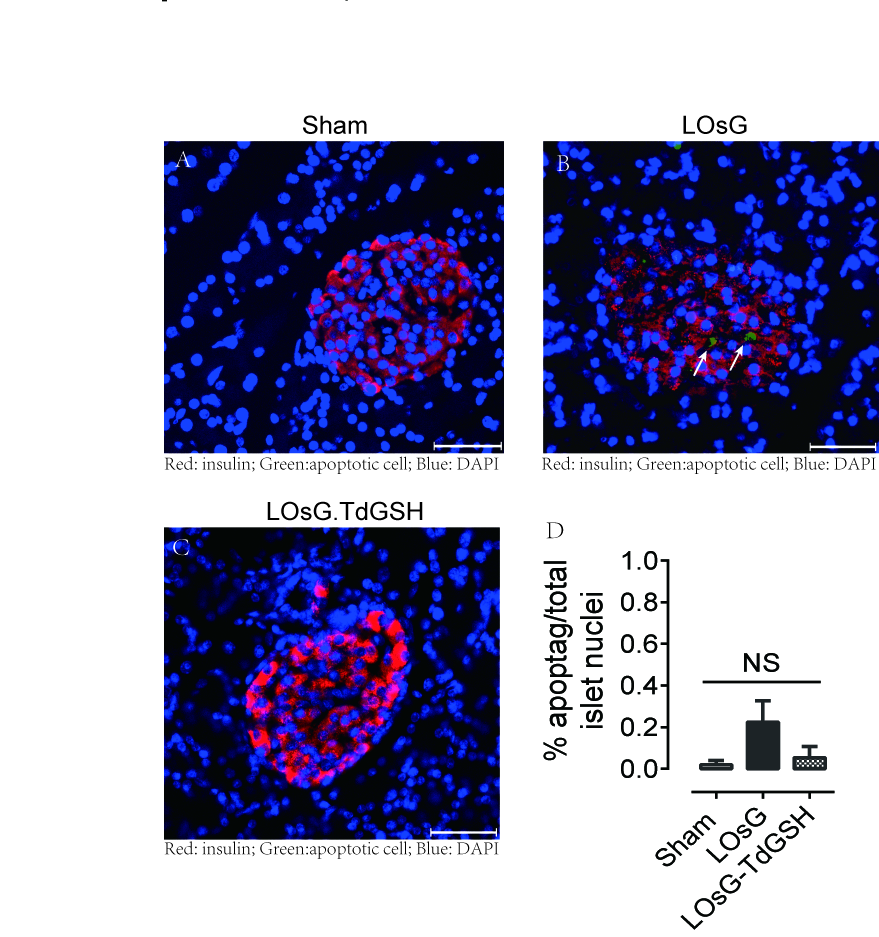

Supplement: Supplementary file 5 — Figure S4 [file 41419_2019_1552_MOESM5_ESM.tif]

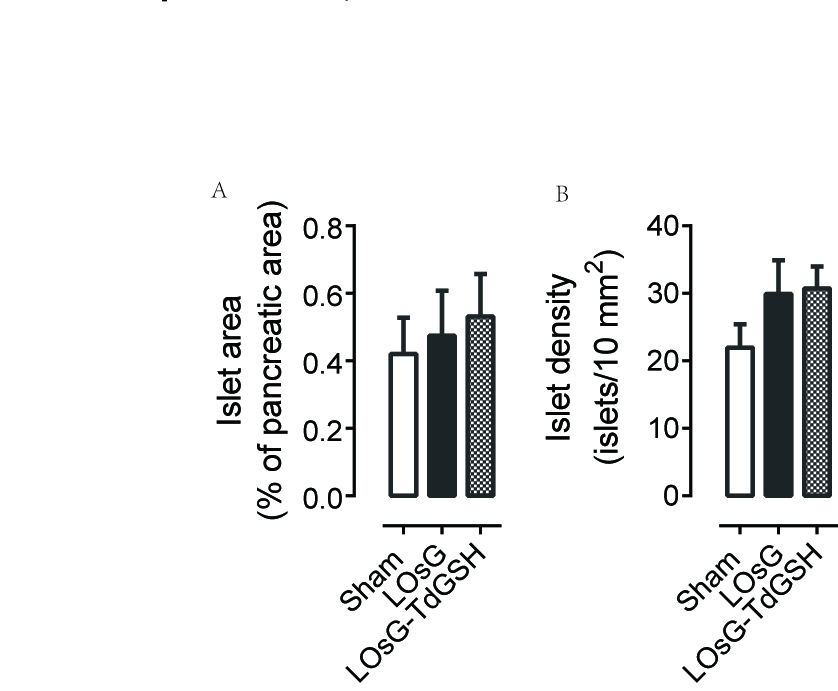

Supplement: Supplementary file 6 — Figure S5 [file 41419_2019_1552_MOESM6_ESM.tif]

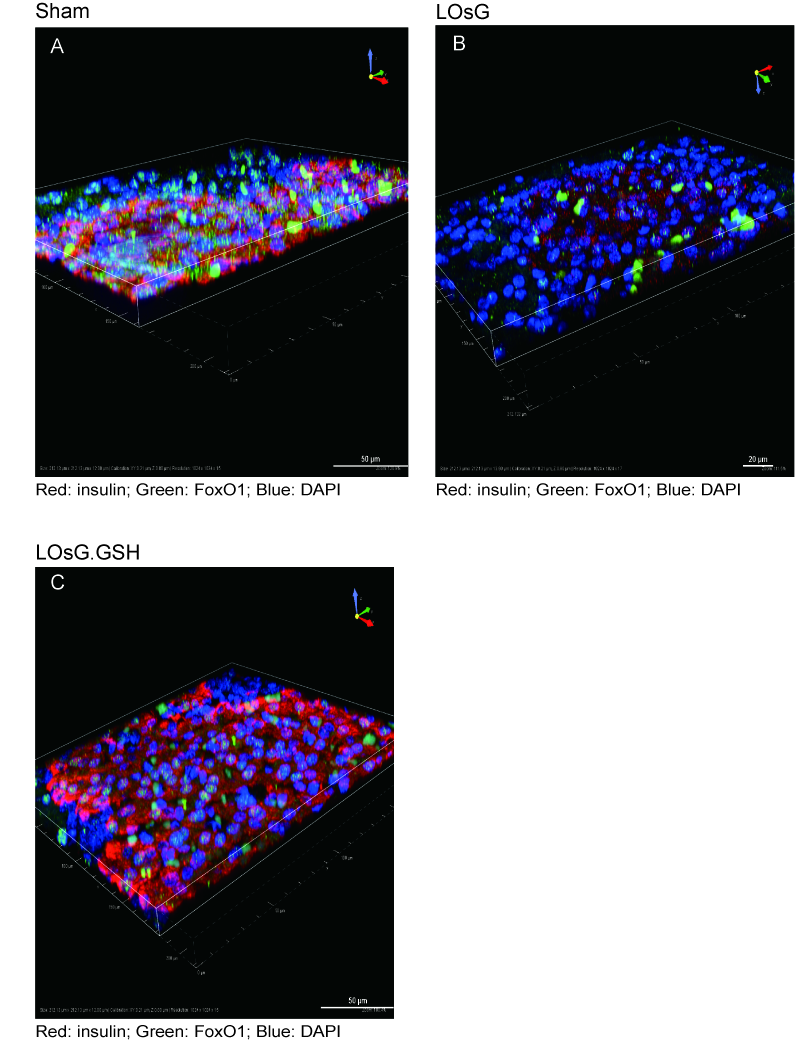

Supplement: Supplementary file 7 — Figure S6 [file 41419_2019_1552_MOESM7_ESM.tif]

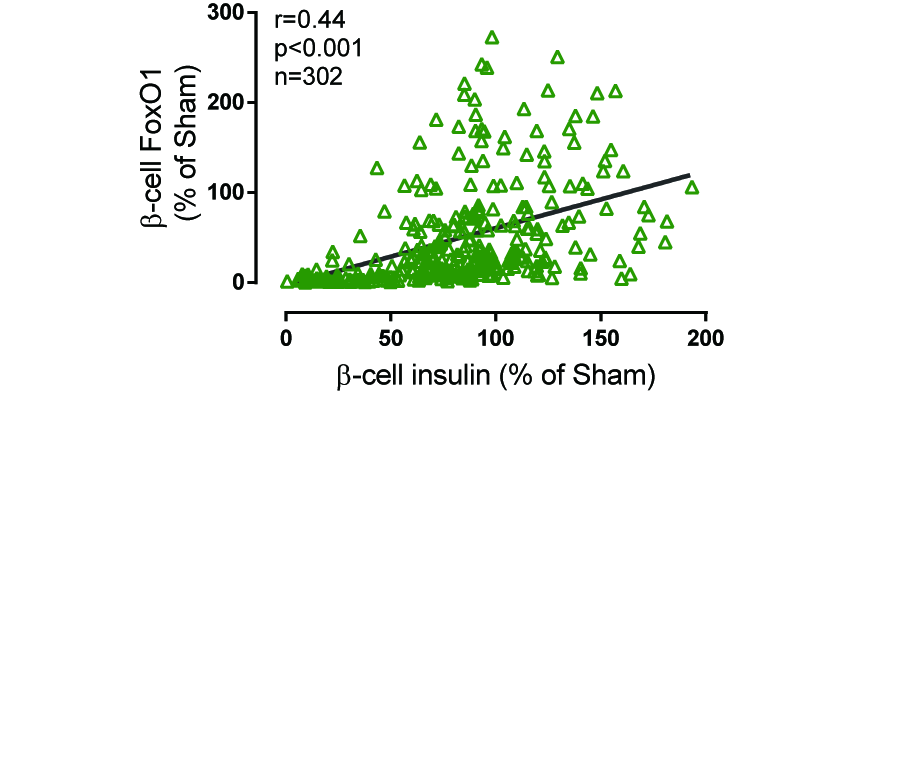

Supplement: Supplementary file 8 — Figure S7 [file 41419_2019_1552_MOESM8_ESM.tif]

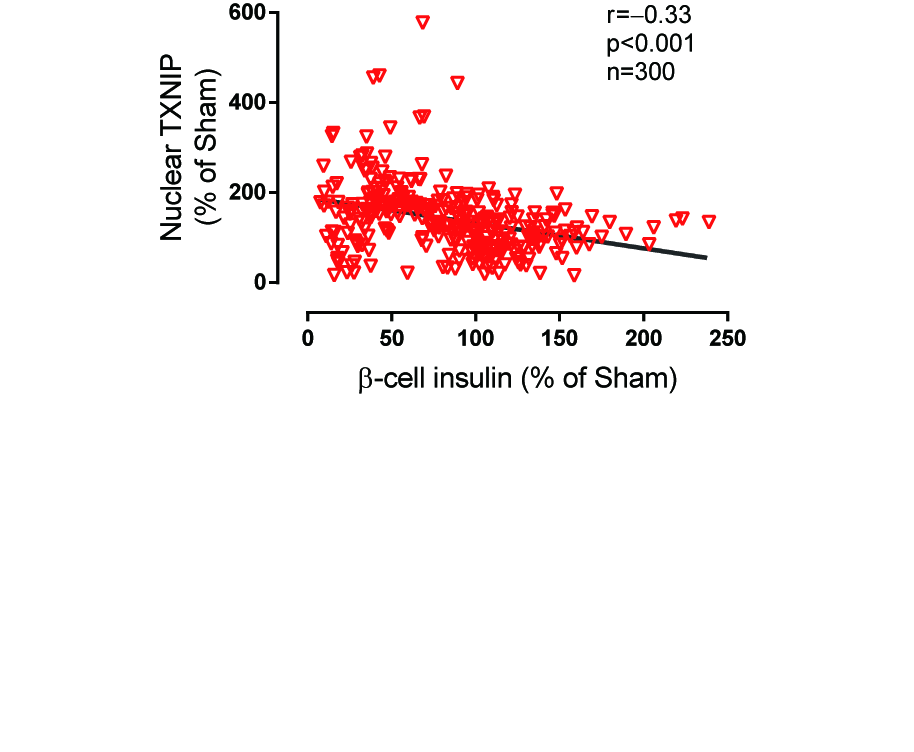

Supplement: Supplementary file 9 — Figure S8 [file 41419_2019_1552_MOESM9_ESM.tif]

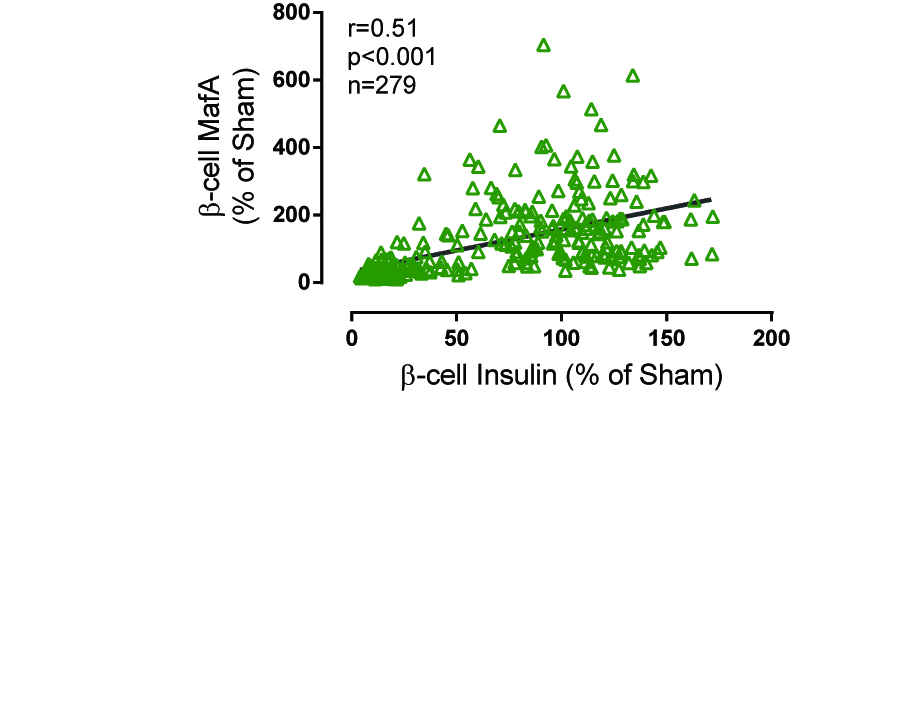

Supplement: Supplementary file 10 — Figure S9 [file 41419_2019_1552_MOESM10_ESM.tif]

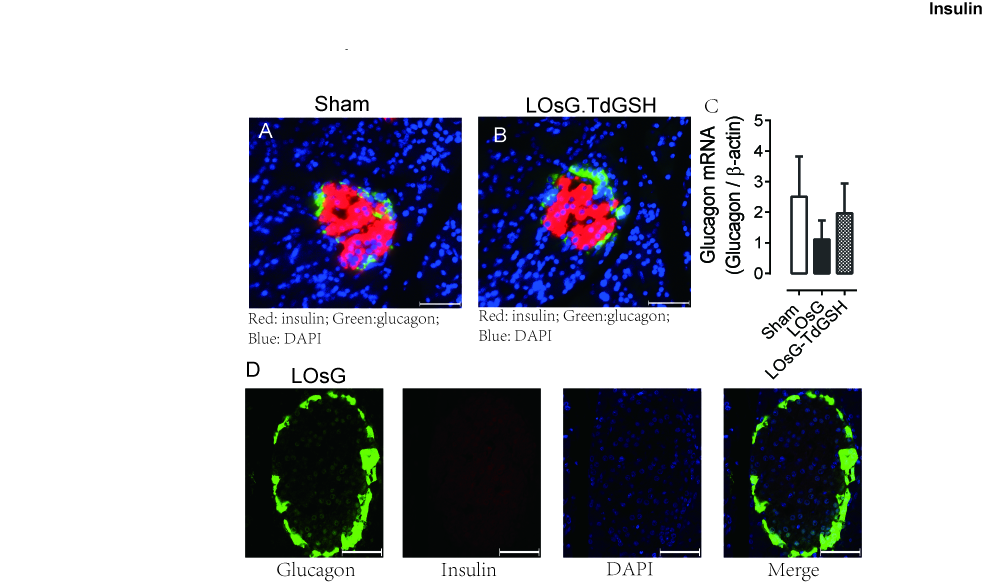

Supplement: Supplementary file 11 — Figure S10 [file 41419_2019_1552_MOESM11_ESM.tif]

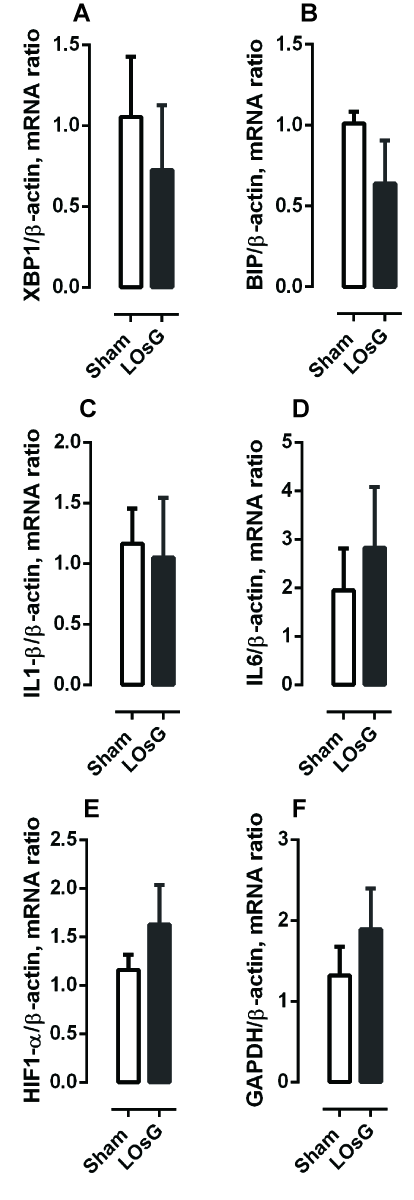

Supplement: Supplementary file 12 — Figure S11 [file 41419_2019_1552_MOESM12_ESM.tif]
